# Glucose Restriction Plus Refeeding in Vitro Induce Changes of the Human Adipocyte Secretome with an Impact on Complement Factors and Cathepsins

**DOI:** 10.3390/ijms20164055

**Published:** 2019-08-20

**Authors:** Qi Qiao, Freek G. Bouwman, Marleen A. van Baak, Johan Renes, Edwin C.M. Mariman

**Affiliations:** Department of Human Biology, NUTRIM School of Nutrition and Translational Research in Metabolism, Maastricht University Medical Centre, 6200 MD Maastricht, The Netherlands

**Keywords:** adipokines, SGBS adipocytes, glucose restriction, in vitro fat regain, weight regain, complement factors, cathepsins, extracellular remodeling

## Abstract

Adipose tissue is a major endocrine organ capable of secreting adipokines with a role in whole-body metabolism. Changes in the secretome profile during the development of obesity is suspected to contribute to the risk of health complications such as those associated with weight regain after weight loss. However, the number of studies on weight regain is limited and secretome changes during weight regain have hardly been investigated. In an attempt to generate leads for in vivo studies, we have subjected human Simpson Golabi Behmel Syndrome adipocytes to glucose restriction (GR) followed by refeeding (RF) as an in vitro surrogate for weight regain after weight loss. Using LC-MS/MS, we compared the secreted protein profile after GR plus RF with that of normal feeding (NF) to assess the consequences of GR plus RF. We identified 338 secreted proteins of which 49 were described for the first time as being secreted by adipocytes. In addition, comparison between NF and GR plus RF showed 39 differentially secreted proteins. Functional classification revealed GR plus RF-induced changes of enzymes for extracellular matrix modification, complement system factors, cathepsins, and several proteins related to Alzheimer’s disease. These observations can be used as clues to investigate metabolic consequences of weight regain, weight cycling or intermittent fasting.

## 1. Introduction

Obesity has become a worldwide critical health issue because it is frequently accompanied by the development of health complications such as type II diabetes, cardiovascular diseases, respiratory problems and certain types of cancer [1]. Obesity is characterized by excess body fat mass, which is mostly stored in the adipose tissue. Additionally, adipose tissue is known as a major endocrine organ capable of secreting various signaling and mediator proteins, termed adipose-derived secreted proteins or adipokines [2]. Studies have shown that the secreted proteins are hormones, cytokines, extracellular matrix related proteins as well as proteins involved in cardiovascular, lipid and glucose metabolism [3]. These adipokines have a variety of effects on homeostasis and metabolism by autocrine, paracrine and endocrine activity, which could contribute to the development of obesity and obesity-associated complications [4,5].

For overweight and obese people, weight loss is an indicated remedy that can reduce the risk for comorbid conditions [6]. A calorie restrictive diet is part of a common practice to try and lose weight. Losing 5% of body weight already results in significant improvement of health parameters such as lower blood pressure, plasma glucose and insulin levels [7]. In parallel, in vitro studies have illustrated that calorie restriction (CR) results in an altered secretion profile of adipocytes, comprised of a less inflammatory phenotype and a reversed expression of detrimental adipokines [8]. However, weight loss is often followed by weight regain. Generally, up to 80% of people who lost weight on CR, regain weight and often return to their original weight or even beyond, within one or two years [9]. Moreover, it has been suggested that weight regain after weight loss could worsen metabolic health [10], possibly mediated by changes in the adipose tissue secretome. Consequently, prevention of weight regain after successful weight loss is a critical challenge for obesity management and it is essential to obtain more knowledge on the causes and consequences of weight regain [11]. So far, the number of biological studies on weight regain has been limited and secretome changes during weight regain are largely unknown.

A growing body of data suggests that Simpson Golabi Behmel Syndrome (SGBS) cells are an excellent tool to study adipokine secretion [3,12,13,14,15]. SGBS cells display a much more active differentiation capacity compared with primary pre-adipocytes in culture, and retain this capacity over at least 30 generations while being similar to primary cells in morphology, physiology and biochemistry [12,13]. Moreover, it has been reported that in vitro differentiated SGBS adipocytes behave similar to human primary adipocytes in functions such as glucose transport, lipogenesis and lipolysis [12,15]. Based on their origin and gene expression comparison, differentiated SGBS cells are regarded as subcutaneous white adipocytes [13]. Therefore, SGBS cells have been widely accepted and used for in vitro adipocyte experiments. Using these cells, we have earlier reported on secretome changes induced by glucose restriction (GR) and found several novel adipokines [8,16]. However, the effect of GR plus refeeding (RF) on adipokine secretion has not been investigated. Recently, we established a protocol for GR followed by RF for SGBS cells and examined the influence on the cellular proteome [17]. Here we report on changes in the in vitro secretome when normal feeding (NF) is compared with GR plus RF in an attempt to obtain leads on possible threats to metabolic health as a consequence of weight regain after weight loss.

## 2. Results

### 2.1. Proteins Secreted from Human SGBS Adipocytes

**Figure 1:**
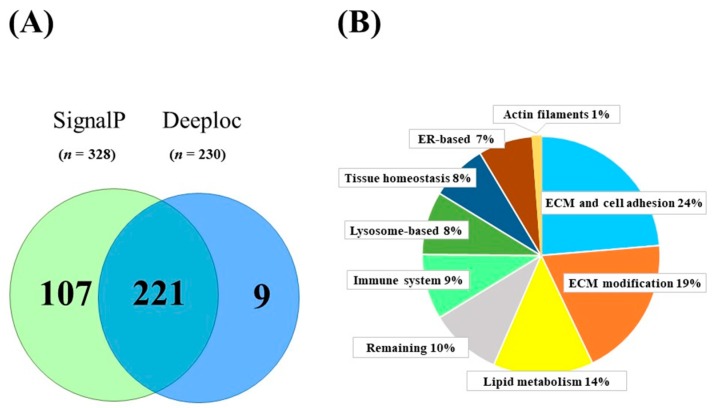
Secreted proteins identified in human Simpson Golabi Behmel Syndrome (SGBS) adipocytes. (**A**) The number of identified secreted proteins by SignalP or Deeploc. (**B**) Pie graph on functional categories of overall identified secreted proteins.

In total, 1326 proteins were identified from the collection medium. By filtering these proteins, SignalP 4.1 detected the presence of a signal peptide in 328 proteins. Deeploc recognized 230 secreted proteins by predicting the extracellular location. In total these two groups represented 337 different proteins (Figure 1A; Table S1). 221 out of 337 proteins both contained a signal peptide and were located in the extracellular space, nine proteins (membrane primary amine oxidase (AOC3), cysteine and glycine-rich protein 1 (CSRP1), galectin-1 (LGALS1), D-dopachrome decarboxylase (DDT), trypsin-3 (PRSS3), metallothionein-1G (MT1G), proteasome inhibitor subunit 1 (PSMF1), ectonucleotide pyrophosphatase/phosphodiesterase family member 2 (ENPP2), small proline-rich protein 2G (SPRR2G)) were only predicted by the extracellular space and 107 proteins only had a signal peptide for secretion (Figure 1A). In addition, we manually checked the list of proteins which were not recognized as secreted by SignalP or Deeploc, for proteins known by their function to be secreted. So far, only complement factor 1Q (C1q) was found to be missed by the programs. Thus, C1q was added to our analysis list with a total of 338 secreted proteins (Table S1).

### 2.2. Newly Identified Secreted Proteins of Human SGBS Adipocytes

Literature on secreted proteins from adipocytes or adipose tissue was searched online and in total 24 research papers and five reviews were found (Table 1) [2,3,8,16,18,19,20,21,22,23,24,25,26,27,28,29,30,31,32,33,34,35,36,37,38,39,40,41,42]. Comparing our secretome data set with the data reported in these papers, 49 secreted proteins were identified as novel secreted proteins that had not been described before for adipocytes or adipose tissue (Table 2). Of these proteins, 46 proteins were annotated as classical secreted proteins (S or S + D in Table 2) and three as non-classical secreted proteins (D in Table 2; MT1G, PSMF1, SPRR2G). 

### 2.3. Functional Categories of Identified Proteins

To get information of the 338 proteins (Table S1) secreted by SGBS adipocytes, functional classification was done according to information on genes/proteins in databases: GeneCards [43], UniProt [44] and PubMed [45]. Generally, these proteins could be classified into nine categories according to biological function with “extracellular matrix (ECM) and cell adhesion” (80 proteins, 24%) and ‘ECM modification’ (65 proteins, 19%) representing the largest groups (Figure 1B). Notably, 12 of the 30 proteins in the immune system category appeared to be complement factors and seven of the 29 proteins in the lysosome-based group were cathepsins.

### 2.4. Adipocyte Secretome Changes after GR Plus RF as Compared with NF

Recently we have reported that the growth rate of fat droplets during NF and during RF after GR shows similar kinetics, which allowed us to investigate the combined influence of GR plus RF on the cellular proteome [17]. In the present study we focused on the secretome of those samples and searched for secreted proteins, of which the abundance was influenced by GR plus RF. For that, protein abundances were quantified by liquid chromatography tandem mass spectrometry (LC-MS/MS) after NF (T18) and after GR plus RF (T22RF). 39 proteins were significantly changed by GR plus RF compared to NF (Table 3) with 18 proteins being up-regulated and 20 proteins being down-regulated. These 39 proteins can be divided into nine functional categories, which seem to parallel the functional categories of the total identified secretome. 13 out of 39 proteins were related to the ECM with seven proteins belonging to the ECM and cell adhesion group and six proteins belonging to ECM modification group. It indicates that GR plus RF induces specific changes to the ECM. Four of the 39 proteins were up-regulated with a FC > 4: complement factor B (CFB), ADAMTS-like protein 1 (ADAMTSL1), target of Nesh-SH3 (ABI3BP), liver carboxylesterase 1 (CES1), and two were down-regulated with a FC > 4: sortilin (SORT1) and dermokine (DMKN). Changes of protein expression during GR plus RF could be due to different mechanisms with major changes either during GR and/or during RF. Therefore, we examined the changes of abundance of the 39 proteins during the separate phases of GR and RF (Table S2). The results confirmed the existence of different regulatory mechanisms. For instance, ADAMTSL1 was up-regulated 10.47× during GR but remained at this level of expression during RF. A similar pattern of expression was observed for prostaglandin-H2 D-isomerase (PTGDS) that was up-regulated 8.44× during GR but only up-regulated 1.51× during RF. ABI3BP did not change during GR, but was 5.04× up-regulated during RF. SORT1 was only slightly down-regulated (1.56×) during GR but 4.07× down-regulated during RF.

## 3. Discussion

In the present study, we performed GR followed by RF of SGBS cells as a simple in vitro surrogate for in vivo weight regain after weight loss and investigated the changes of human adipocyte-derived secreted proteins. Comparison between NF and GR plus RF was made to gain information on changes induced to the secretome that could serve as leads to get further insight into the consequences of weight regain for metabolic health. Our results show that GR plus RF induced adipocyte secretome changes involving biological pathways of ECM remodeling, lipid metabolism, complement system, and tissue homeostasis. Furthermore, 49 secreted proteins were described here for the first time as being secreted by adipocytes. 

Harvesting secreted proteins from the culture medium inevitable leads to contamination with leaked cellular proteins. We have applied bioinformatics analysis to identify the secreted proteins either as classical secreted proteins, which are typically targeted to the endoplasmic reticulum by a signal peptide, and non-classical secreted proteins without a signal peptide. Till now, SignalP [46] has been a well-accepted method for sorting classical secreted proteins and SecretomeP [47] has been widely used for non-classical secretion. When we first used SignalP in combination with SecretomeP, this yielded 739 potentially secreted proteins in the current study. However, recently Henrik et al. [48] reported that SecretomeP induces more than 20% false positives. Instead, Deeploc obtained the highest accuracy (78% for subcellular localization; 92% for membrane-bound or soluble) on predicting non-classical secreted proteins when compared with other methods [49]. Therefore, in the current study we used the combination of SignalP and Deeploc, which led to 337 secreted protein candidates, of which 66% (221) were ranked as secreted by both programs. Yet, it should be noted that even with our stringent method of selection, a certain level of misclassification cannot be avoided. Additionally, some proteins may be aberrantly classified as non-secreted as observed for complement factor C1q.

The largest change in abundance induced by the combined effects of GR plus RF was the 9.57 × up-regulation of ADAMTSL1. This is a metalloproteinase located in the ECM and known to degrade aggrecan [50]. Two other metalloproteinases (MMP2 and MMP8) are up-regulated as well, suggesting that after GR plus RF, the ECM is in a catabolic state. Moreover, three proteins involved in the maturation of collagens, P4HA1, PPIC and SERPINH1, are down-regulated. Recently we have shown that inside the cells GR leads to an upregulation of certain focal adhesion proteins [17]. It suggests that upon GR plus RF adipocytes intensify the cell-cell interaction while going through a phase of increased ECM flexibility. At the moment, it is not clear whether this also occurs in vivo and whether it would influence weight regain or the metabolic condition after weight regain. Still, in a previous weight loss/follow-up intervention study, expression of the *ADAMTSL1* and *MMP2* genes were significantly up-regulated (FC = 1.09, q = 0.05; FC = 1.24, q < 0.0001, respectively) four weeks after return to a balanced diet [51]. This indicates that ECM adaptations occur also in vivo during weight loss and weight regain.

**Figure 2:**
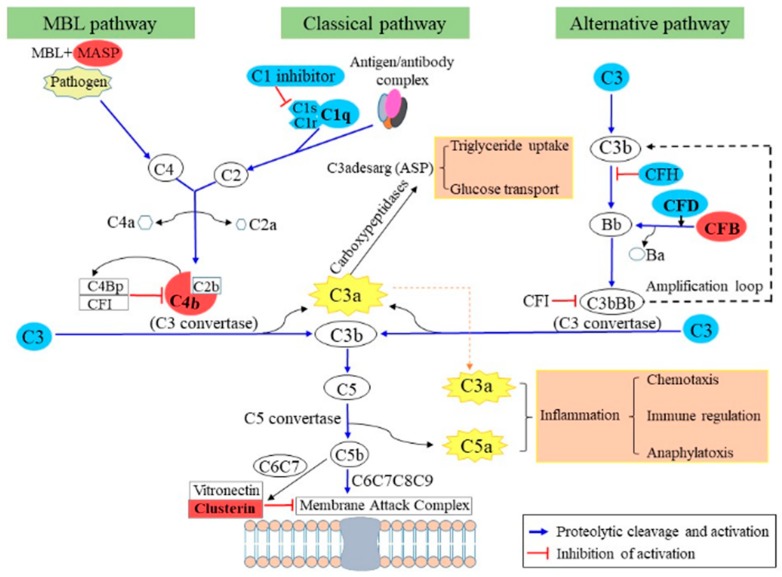
Complement activation pathway comparing GR plus RF with NF. Secreteome changes during GR plus RF vs NF indicate the up regulation of MBL and alternative pathways of the complement system, which may trigger the uptake of triglycerides and glucose by adipocytes on one hand and promote inflammation on the other, which both may have an effect on health after weight regain. Proteins in blue means that the expression was down-regulated (*p* < 0.1), red means up-regulated (*p* < 0.1), white are proteins that were not detected in our results. RF: refeeding, NF: normal feeding, GR: glucose restriction. MBL: mannose-binding lectin. MASP: mannose-binding lectin serine protease.

It is well established that adipose tissue secretes various components of the complement system. During development of overweight and obesity, the secreted levels of complement factors change, which is thought to contribute to the chronic low level inflammation of the adipose tissue and the development of health complications such as insulin resistance, type II diabetes and cardiovascular disorders [1]. In this respect, it has been suggested that modulation of the complement system could be a target for the prevention and therapy of obesity-associated metabolic diseases [11,52,53,54,55]. Twelve proteins with an influence on complement activation were identified in our in vitro system and eight of them were complement factors (Table 4). Of those proteins, complement factor B (CFB) and complement factor 4B (C4-B) were significantly increased by GR plus RF, whereas the abundances of C1q and complement factor D (CFD) were significantly reduced. Such changes in vivo after weight loss and weight regain might lead to systemic changes in complement activity, especially in people with a high fat mass [52]. More generally, it could have local effects in the adipose tissue itself. Reduction of C1q and of C1s (*p* = 0.13) indicates a lower classical complement pathway. Yet, the increase of C4-B and mannan-binding lectin serine protease 1 (MASP1) suggest an increase of the C3 convertase C4bC2b. Together with the strong increase of CFB, a local increase of C3a and C3b could be expected. C3a can be converted to C3desArg (acylation-stimulating protein, ASP) by carboxypeptidases B and N [56]. Here we found that adipocytes secrete carboxypeptide E, which is similarly able to remove arginine from the carboxyterminal tail of proteins. An increase of C3desArg would stimulate triglyceride uptake and glucose transport into adipocytes [57]. In the mouse it has been shown that CFB promotes adipocyte maturation and growth of fat droplets [58]. It is tempting to speculate that GR plus RF could have the same consequence. In addition, C3a has immune-modulatory properties and is often regarded as a pro-inflammatory factor [59]. Factor C3b in interaction with CBb converts C5 into C5a and C5b. C5a has chemotaxis activity and attracts neutrophils, basophils and macrophages. C5b forms with C6–C9 the membrane attack complex, which could be counteracted by the increase of clusterin (FC = 1.40, *p* = 0.09) [60]. Altogether, our in vitro observations suggest that changes in the complement system through GR plus RF may trigger uptake of triglycerides and glucose by adipocytes and may promote the attraction of immune cells (Figure 2). In fact, in the present study triglyceride content was measured by ORO staining and by measuring the diameter of the five biggest fat droplets [17]. The OD value was higher by about 10%, being 1.55 after NF and 1.69 after GR plus RF (*p* = 0.13). In line, the diameter was higher by about 14%, being 1.13 µm after NF and 1.29 µm after GR plus RF (*p* = 0.02). Although this is keeping with the proposed hypothesis, we have no absolute proof to attribute the increased fat content to the influence of complement factors. Regarding the involvement of the innate immune cells, gene expression studies in vivo have indicated that poor ability to reduce myeloid activity from the adipose tissue after weight loss is associated with increased risk of weight regain [11,61].

Various lysosome-based proteins were identified by our secretome profiling (Table 4). Lysosomes function as a key degradative compartment of cells, of which lysosomal cathepsins display an essential role in maintaining cell homeostasis by autophagy and extracellular matrix degradation [62,63,64]. Their extracellular function is mostly associated with pathology and disease including metabolic syndrome in people with obesity [65,66]. In the current study, we observed seven different cathepsins (A, B, D, F, K, L and Z) secreted by adipocytes. Five of the identified cathepsins were up-regulated by trend (Table 4) and two cathepsins were significantly up-regulated (Table 3 and Table 4) after GR plus RF versus NF. In detail, cathepsin A (CTSA) and cathepsin L (CTSL) were significantly increased while other cathepsins showed a trend (Table 4). CTSA is able to stabilize the extracellular beta-galactosidase/neuraminidase-1 complex, which is involved in the formation of elastic fibers [67]. In addition, it processes important vascular proteins including endothelin-1, bradykinin and angiotensin I, which are important for the regulation of blood pressure [68]. Pharmacological inhibition indicates a role of CTSL in adipogenesis/fat storage and glucose tolerance. Inhibition of CTSL reduced fibronectin degradation and increased the levels of the beta-subunits of the insulin receptor and insulin-like growth factor-1-receptor [69]. Studies have also shown that CTSL, like cathepsin S (CTSS), has proatherogenic properties [70]. However, those studies did not specifically look at the extracellular function of CTSL. Yet, a literature survey has shown that CTSL is one of the cathepsins involved in extracellular matrix degradation and tissue remodeling [63]. Serum levels of CTSL did not alter following 6-month CR in obese women [71], but were reduced after an 8 week lifestyle intervention [72]. Here we show that GR plus RF significantly increased the extracellular CTSA and CTSL level. It seems therefore warranted to study the in vivo consequences of weight regain on plasma levels of cathepsins and assess the influence on fat storage, blood pressure and glucose tolerance.

A number of proteins that change abundance significantly between GR plus RF and NF, has been described in relation to Alzheimer’s disease. Amyloid-beta A4 protein (APP), which is a major component of the amyloid plaques in the brain of Alzheimer’s patients [73], is 1.43× down-regulated. Extracellular SORT1, which has been reported to be involved in plaque formation [74], is 5.13× down-regulated in our study. PTGDS was 3.67× up-regulated due to GR plus RF. Interestingly, an up-regulation of the gene for *PTGDS* after ingestion of soybeans could lead to reduced amyloid-β accumulation and improved cognition [75]. A fourth protein, chitinase-3-like protein 2 (CHI3L2) is 3.43 × up-regulated as the consequence of GR plus RF. CHI3L2 is known to bind to glycans, but lacks the required domain for chitinase activity [44]. Recently, it has been reported that the regional expression of CHI3L2 in the brain of late-onset Alzheimer’s patients is altered in comparison to healthy persons [76], but its function is presently unknown. Together, it would be worth to investigate whether weight cycling in humans could influence the expression of those proteins as well.

It should be noted that there are limitations to the present study. Firstly, SGBS cells have widely been used in vitro as a substitute for human white subcutaneous adipocytes since 2001 [13], but a study by Yeo et al. [77] indicated that SGBS cells may also have browning potential. Secondly, arguments including the fact that MS data are based on multiple peptide quantifications per protein, make it convincing that MS quantification is highly accurate [78,79]. Validation by another method has the risk of devaluating the quantitative data. Therefore, such validation was not performed. Thirdly, an in vitro model for weight loss and regain preferably is based on ‘obese’, hypertrophic fat cells. However, a hypertrophic phenotype of adipocytes differentiated in culture is difficult to define. For the present model it can be said that after 12 days of differentiation, SGBS cells contain 4× as much triglycerides than differentiated primary adipocytes [77]. Here we used 14 days differentiated SGBS cells with a 24% higher diameter of the five biggest fat droplets as compared to day 12 indicating an even higher fat content on day 14. Therefore, we feel confident to regard our findings as valuable clues for the consequences of weight loss and regain in overweight people. This can now be examined in human intervention studies.

## 4. Materials and Methods

### 4.1. Cell Culture

Human SGBS cells were obtained from Prof. Dr. M. Wabitsch (University of Ulm, Germany) [12]. SGBS pre-adipocytes were cultured in 6-well plates (Corning, Sigma-Aldrich, Zwijndrecht, The Netherlands) with Gibco™ Dulbecco’s Modified Eagle Medium: Nutrient Mixture F-12 (DMEM/F-12, 1:1) media supplemented with 66 mmol/L biotin, 34 mmol/L D-pantothenate, 10% fetal calf serum (Bodinco BV, Alkmaar, The Netherlands) and 1% penicillin and streptomycin (Life Technologies, Thermo Fisher Scientific, Bleiswijk, The Netherlands) as described before [17]. Differentiation started once pre-adipocytes reached 90% confluence. During two weeks of differentiation (T0–T14), SGBS cells first went through four days with quick differentiation medium (serum-free DMEM/F12 medium containing 2 mg/mL human transferrin, 200 µmol/L human insulin, 5 mmol/L Cortisol, 20 µmol/L triiodothyronine, 1 mmol/L 3-isobutyl-1-methylxanthine and 5 mmol/L rosigilitazone). The other 10 days cells remained in 3FC medium (serum-free DMEM/F12 medium containing 2 mg/mL human transferrin, 200 µmol/L human insulin, 5 mmol/L Cortisol, 20 µmol/L triiodothyronine) as described before [17]. All chemicals were purchased from Sigma (Sigma-Aldrich, Zwijndrecht, The Netherlands) unless otherwise stated.

For GR, mature adipocytes on day 14 (T14) were cultured in basic DMEM/F12 medium (without glucose and phenol red (Cell Culture Technologies, Gravesano, Switzerland)), supplemented with 20 nmol/L human insulin and 0.1 mmol/L D-glucose for 96 h (T18GR). As the feeding control, mature adipocytes at T14 originating from the same pre-adipocytes were cultured for 96 h in NF medium (T18): DMEM/F12 medium without glucose and phenol red, but supplemented with 20 nmol/L human insulin and 17.5 mmol/L D-glucose.

**Figure 3:**
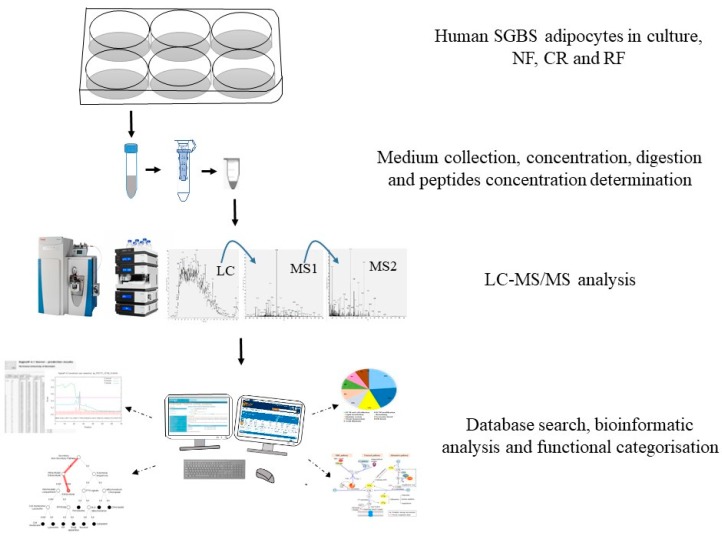
Workflow of the secretome profiling. Firstly, human SGBS adipocytes were cultured and after NF, GR and RF, medium was collected for all the time points. After sample preparation, proteins were identified and quantified by LC-MS/MS. Finally, bioinformatic and statistic analysis were performed for functional interpretation. NF: normal feeding, GR: glucose restriction, RF: refeeding.

For RF, after 96 h of GR, the cells were transferred to DMEM/F12 medium without glucose and phenol red (Cell Culture Technologies) but supplemented with 20 nmol/L human insulin and 17.5 mmol/L D-glucose for another 96 h (T22RF). From T14 onwards, the medium was gently refreshed every second day. Figure 3 provides an overview of the experimental approach.

### 4.2. Collection of Secretion Medium

In order to avoid interference of phenol red with MS, for collecting the secretome at T14, adipocyte medium was changed to collection medium: basal medium (DMEM/F12 (1:1) without glucose and phenol red (Cell Culture Technologies, Gravesano, Switzerland)), supplemented with 20 nmol/L human insulin and 17.5 mmol/L glucose for 48 h (T12–T14). The collection medium at T14 was collected in a separate vial for each well. For NF, GR and RF, the medium was already without phenol red from T14 onwards. To collect the secretome after NF, GR and RF, the medium was collected at day 18 (T18 and T18GR) and day 22 (T22RF) from each well separately. The collected medium (4 mL per well) was centrifuged at 5000 rpm for 10 min (Universal 30 RF, Hettich Benelux B.V., The Netherlands). Thereafter, the supernatant was gently moved to a new tube. The whole experiment was performed three times with triplicate samples. The first time the experiment was performed, the triplicate medium samples per time point were pooled to serve proper protocol assessment. For the second and third time that the experiment was performed, the triplicate samples were kept separately. This provided seven replicate samples per time point with in total 28 samples. These were snap-frozen in liquid nitrogen and stored at −80 °C for further analysis.

### 4.3. Sample Preparation

All samples were treated similarly according to the FASP protocol as described by Wisniewski et al. [80]. In short, after thawing and brief vortexing, the medium of each vial was added to a pre-rinsed filter device (Amicon^®^ Ultra−4 Centrifugal Filter Units, Sigma-Aldrich, Germany), centrifuged at 4000× *g* at 20 °C for 30 min. The fluid was discarded and the concentrated protein sample on the filter was washed with 3.5 mL 50 mmol/L ammonium bicarbonate and centrifuged at 4000× *g* at 20 °C for 30 min. For reduction, 15 µL of 200 mmol/L dithiothreitol was added and the filter was incubated at room temperature for 45 min. Next, to accomplish alkylation 18 µL of 400 mmol/L iodoacetamide solution was added and the filter was incubated at room temperature in the darkness for another 45 min. To stop the alkylation, 30 µL of 200 mmol/L dithiothreitol was added and the filter was incubated at room temperature for 45 min.

The alkylated protein sample on the filter was washed with 50 mmol/L ammonium bicarbonate at 4000× *g* at 20 °C for 40 min. To each concentrated protein sample 3 µg trypsin/Lys-C Mix was added, and after gentle mixing the filter device was incubated at 37 °C overnight. Peptide concentration was measured by the Pierce Quantitative Colorimetric Peptide Assay according to the manufacture’s protocol (Thermo Fisher Scientific (#: 23275), Bleiswijk, The Netherlands). Then, digested peptides were diluted to the same final concentration of 0.25 μg/μL by 50 mmol/L ammonium bicarbonate.

### 4.4. Label-Free Protein Identification and Quantification

The mass analysis was performed using a nanoflow HPLC instrument (Dionex ultimate 3000, Thermo Fisher Scientific, Bleiswijk, The Netherlands), which was coupled on-line to a Q Exactive mass-spectrometer (Thermo Fisher Scientific, Bleiswijk, The Netherlands) with a nano-electrospray Flex ion source (Proxeon, Thermo Fisher Scientific, Bleiswijk, The Netherlands). An equal amount of Pierce Digestion Indicator peptides was added to all peptide samples as internal standard. 5 μL of this mixture was loaded onto a C18-reversed phase column (Acclaim PepMap C18 column, 75 μm inner diameter × 15 cm, 2 μm particle size, Thermo Fisher Scientific, Bleiswijk, The Netherlands). The peptides were separated with a linear gradient of 4–68% buffer B (80% acetonitrile and 0.08% formic acid) at a flow rate of 300 nL/min for 120 min.

MS data were acquired using a data-dependent top-10 method, dynamically choosing the most abundant precursor ions from the survey scan (280–1400 m/z) in positive mode. Survey scans were acquired at a resolution of 70,000 and a maximum injection time of 120 ms. The dynamic exclusion duration was 30 s. Isolation of precursors was performed with a 1.8 m/z window and a maximum injection time of 200 ms. The resolution for HCD spectra was set to 17,500 and the normalized collision energy was 30 eV. The under-fill ratio was defined as 1.0%. The instrument was run with peptide recognition mode enabled, but exclusion of singly charged and charge states of more than five. The entire experiment was repeated three times.

### 4.5. Database Search and Quantification

The MS data were searched using Proteome Discoverer 2.2 Sequest HT search engine (Thermo Fisher Scientific, Bleiswijk, The Netherlands) based on the UniProt human database [81]. The false discovery rate was set to 0.01 for proteins and peptides, which had to have a minimum length of six amino acids. The precursor mass tolerance was set at 10 ppm and the fragment tolerance at 0.02 Da. One miss-cleavage was tolerated, oxidation of methionine was set as a dynamic modification as well as carbamidomethylation of cysteines. Label free quantitation was conducted using the Minora Feature Detector node in the processing step and the Feature Mapper node combined with the Precursor Ions Quantifier node in the consensus step with default settings within Proteome Discoverer 2.2.

### 4.6. Data Normalization

The LC-MS-analysis was done in seven runs, each run containing a sample from each time point (T14, T18, T18GR and T22RF). Data normalization was done in two steps.

First, to correct data for possible differences between runs, we chose the 426 proteins, which were present in all of the test samples. We calculated the mean abundance of those 426 proteins in all seven runs (M) and mean abundance of those 426 proteins per run (m_x_ for run *x*). Normalization factor 1 for run *x* (f1*_x_*) = M ÷ m*_x_*. Data were firstly corrected (D1) as follows: D1 = f1*_x_* × original tested protein abundance in run *x*.

Additionally, the Pierce Indicator added to each sample was normalized by f1. The second normalization was then performed to stratify the protein abundances according to the Pierce Indicator. Normalization factor 2 for sample *y* (f2*_y_*) = Pierce’s mean abundance from all 28 samples ÷ Pierce abundance in sample *y*. In general, the second normalization step was: D2 = f2 × D1. More specifically, the abundance of a protein in sample y of run x was normalized as f1*_x_* × f2*_y_* × original tested protein abundance.

### 4.7. Validation of Secreted Proteins and Imputation of Missing Values

To verify the secreted nature of the identified proteins, their amino acid sequences were obtained from UniProt and analyzed with SignalP and Deeploc. SignalP 4.1 Server [46,82] was used to recognize classical secreted proteins by the fact that they contain a signal peptide for secretion, Deeploc−1.0 [49] was used for prediction of the subcellular localization by creating a recurrent neural network relying on protein sequence information per se. Proteins containing a signal peptide or located in the extracellular space were chosen as secreted proteins for further analysis.

Performing LC-MS analysis of proteins, values could be missing for various reasons [83]. When per time point three or less of the seven samples had missing values, the Multiple Imputation routine of SPSS was used to impute those values. Finally, only proteins recognized as secreted and with no more than three missing values were used for further analysis.

### 4.8. Statistical Analysis

Data were described as mean ± SEM, variable abundances were log_2_-transformed. To determine possible effects over time between NF and RF after GR, two-tailed dependent T-test was carried out with a cut-off for significance of *p* < 0.05. Statistical analyses were conducted using SPSS (version 22.0 Chicago, Illinois, USA). Fold changes (FC) from T18 to T22RF were calculated as follows: FC = 2 (log_2_ T22RF − log_2_ T18).

## 5. Conclusions

In summary, in this study we reported for the first time 94 proteins being secreted by adipocytes. In addition, our in vitro study demonstrated that GR followed by return to NF leads to changes in the secretome of adipocytes in comparison with NF alone. Major changes are related to extracellular matrix modification, factors of the complement system, extracellular cathepsins, and several proteins relevant for Alzheimer’s disease. These observations can now be used as clues to investigate the metabolic consequences of weight regain, weight cycling or intermittent fasting.

## Figures and Tables

**Table 1:** Literature Reports on Adipocyte Adipokine Profiling.

Order	Reference	Refs	Source	Secreted	Novel
1	Wang et al. (2004)	[18]	Mice (3T3L1 cells)	26	15
2	Chen et al. (2005)	[19]	Rat fat pad	84	53
3	Molina et al. (2009)	[20]	Mice (3T3L1 cells)	147	NA
4	Celis et al. (2005)	[21]	Human adipocytes	359	NA
5	Mutch et al. (2009)	[22]	Human primary preadipocytes	213	NA
6	Alvarez-Llamas et al. (2007)	[23]	Human visceral fat tissue	108	68
7	Zvonic et al. (2007)	[24]	Human adipose-derived stem cells	101	NA
8	Lim J.M. et al. (2008)	[31]	3T3L1 cell line; primary rat adipocytes	97; 203	54; 132
9	Roelofsen H et al. (2009)	[32]	Human omental tissue (control; test)	155; 141	NA
10	Kim et al. (2010)	[25]	Human subcutaneous adipose tissue	307	NA
11	Rosenow et al. (2010)	[3]	Human SGBS cells	80	6
12	Zhong et al. (2010)	[26]	Human adipocytes	420	107
13	Lee M.J. et al. (2010)	[27]	Human adipose tissue derived stem cells	142	NA
14	Rosenow A. et al. (2012)	[16]	Human SGBS cells	40	2
15	Lehr S. et al. (2012)	[28]	Human primary adipocytes	263	44
16	Roca-Rivada A. et al. (2011)	[40]	Rats (visceral; subcutaneous; gonadal fat)	188; 85; 91	NA
17	Sano S. et al. (2014)	[41]	Mice (3T3L1 cells)	231	NA
18	Roca-Rivada A. et al. (2015)	[38]	Human adipose tissue (visceral; subcutaneous)	136; 64	NA
19	Hartwig S. et al. (2018)	[36]	Human adipocytes	884	67
20	Laria A.E. et al. (2018)	[37]	Mice (3T3L1 cells)	839	80
21	Renes J. et al. (2014)	[8]	Human SGBS cells	57	6
22	Li Z.Y. et al. (2014)	[29]	Human SAT/ VAT	NA	1
23	Ojima K et al. (2016)	[34]	Mice (3T3L1 cells)	74	NA
24	Ali Khan et al. (2018)	[30]	Mice primary adipocytes	499	NA
25	Mariman et al. (2010)	[33]	(Review)_Human and rodent adipocytes	NA	NA
26	Lehr S; et al. (2012)	[2]	(Review)_Human adipocytes	928	NA
27	Renes J. et al. (2013)	[35]	(Review)_Human and rodent adipocytes	NA	NA
28	Pardo M. et al. (2012)	[39]	(Review)_Human and rat adipocytes	NA	NA
29	Lee M.W. et al. (2019)	[42]	(Review)_Human and rat adipocytes	NA	NA

The number in the “secreted” column refers to the number of identified adipocyte secreted proteins, the “novel” column shows the number of newly reported adipokines in that report. NA: there is no exact number for secreted proteins mentioned in the article or supplemental materials.

**Table 2:** List of 49 novel adipocyte secreted proteins.

Order	S or D	UniProt	Gene Symbol	Protein Name
1	S	O00763	ACACB	Acetyl-CoA carboxylase 2
2	S + D	P04745	AMY1A	Amylase, Alpha 1A (Salivary)
3	S	O43570	CA12	Carbonic anhydrase 12
4	S	P55287	CDH11	Cadherin-11
5	S	P19022	CDH2	Cadherin-2
6	S + D	Q9BWS9	CHID1	Chitinase domain-containing protein 1
7	S	P26992	CNTFR	Ciliary neurotrophic factor receptor subunit alpha
8	S + D	Q9UI42	CPA4	Carboxypeptidase A4
9	S + D	O75629	CREG1	Protein CREG1
10	S + D	O00602	FCN1	Ficolin-1
11	S	Q10471	GALNT2	Polypeptide N-acetylgalactosaminyltransferase 2
12	S	P23434	GCSH	Glycine cleavage system H protein, mitochondrial
13	S + D	P06280	GLA	Alpha-galactosidase A
14	S + D	Q9UJJ9	GNPTG	N-acetylglucosamine-1-phosphotransferase subunit gamma
15	S	O75487	GPC4	Glypican-4
16	S + D	P35475	IDUA	Alpha-L-iduronidase
17	S + D	P08476	INHBA	Inhibin beta A chain
18	S + D	Q96I82	KAZALD1	Kazal-type serine protease inhibitor domain-containing protein 1
19	S	Q6GTX8	LAIR1	Leukocyte-associated immunoglobulin-like receptor 1
20	S	P38571	LIPA	Lysosomal acid lipase/cholesteryl ester hydrolase
21	S	O75197	LRP5	Low-density lipoprotein receptor-related protein 5
22	S	Q8ND94	LRRN4CL	LRRN4 C-terminal-like protein
23	S	Q5JRA6	MIA3	Transport and Golgi organization protein 1 homolog
24	S + D	P22894	MMP8	Neutrophil collagenase
25	D	P13640	MT1G	Metallothionein-1G
26	S + D	P41271	NBL1	Neuroblastoma suppressor of tumorigenicity 1
27	S	Q04721	NOTCH2	Neurogenic locus notch homolog protein 2
28	S + D	P48745	NOV	Protein NOV homolog
29	S + D	O95897	OLFM2	Noelin-2
30	S + D	Q8NBP7	PCSK9	Proprotein convertase subtilisin/kexin type 9
31	S	P50897	PPT1	Palmitoyl-protein thioesterase 1
32	S + D	P42785	PRCP	Lysosomal Pro-X carboxypeptidase
33	S + D	P07477	PRSS1	Trypsin-1
34	D	Q92530	PSMF1	Proteasome inhibitor subunit 1
35	S	P10586	PTPRF	Receptor-type tyrosine-protein phosphatase F
36	S	Q15274	QPRT	Nicotinate-nucleotide pyrophosphorylase [carboxylating]
37	S	Q6NUM9	RETSAT	All-trans-retinol 13,14-reductase
38	S + D	O00584	RNASET2	Ribonuclease T2
39	S	Q9H173	SIL1	Nucleotide exchange factor SIL1
40	S	Q99523	SORT1	Sortilin
41	D	Q9BYE4	SPRR2G	Small proline-rich protein 2G
42	S + D	P10124	SRGN	Serglycin
43	S + D	A1L4H1	SSC5D	Soluble scavenger receptor cysteine-rich domain-containing protein SSC5D
44	S	Q8NBK3	SUMF1	Sulfatase-modifying factor 1
45	S	Q8NBJ7	SUMF2	Sulfatase-modifying factor 2
46	S	Q5HYA8	TMEM67	Meckelin
47	S + D	Q8WUA8	TSKU	Tsukushin
48	S	Q06418	TYRO3	Tyrosine-protein kinase receptor TYRO3
49	S	P98155	VLDLR	Very low-density lipoprotein receptor

S: secreted proteins identified by SignalP, D: identified by Deeploc, S + D: identified by both software packages.

**Table 3:** Proteins significantly different between GR plus RF and NF.

Order	Category	Gene Symbol	Accession	Description	T18-T22RF	
					FC_(GR+RF)/NF	*p* Value
1	Actin filaments	GSN	P06396	Gelsolin	−1.73	0.007
2	Complement factors	C1Q	Q07021	Complement 1q subcomponent	−2.94	0.030
3		C4B	P0C0L5	Complement C4-B	2.85	0.002
4		CFB	P00751	Complement factor B	4.06	0.018
5		CFD	P00746	Complement factor D	−1.93	0.002
6	ECM and cell adhesion	CDH13	P55290	Cadherin-13	−2.06	0.044
7		COL15A1	P39059	Collagen alpha-1(XV) chain	1.83	0.012
8		COL5A3	P25940	Collagen alpha-3(V) chain	−1.69	0.047
9		LUM	P51884	Lumican	−1.48	0.025
10		MCAM	P43121	Cell surface glycoprotein MUC18	−1.91	0.028
11		NRCAM	Q92823	Neuronal cell adhesion molecule	−2.39	0.046
12		SERPINE2	P07093	Glia-derived nexin	1.58	0.020
13	ECM modification	ADAMTSL1	Q8N6G6	ADAMTS-like protein 1	9.57	0.006
14		MMP2	P08253	72 kDa type IV collagenase	1.48	0.010
15		MMP8	P22894	Neutrophil collagenase	3.72	0.001
16		P4HA1	P13674	Prolyl 4-hydroxylase subunit alpha-1	−1.80	0.004
17		PPIC	P45877	Peptidyl-prolyl cis-trans isomerase C	−1.47	0.012
18		SERPINH1	P50454	Serpin H1	−2.19	0.001
19	ER-based	CALU	O43852	Calumenin	−2.36	0.002
20		HYOU1	Q9Y4L1	Hypoxia up-regulated protein 1	1.78	0.009
21		RCN1	Q15293	Reticulocalbin-1	−2.23	0.002
22		TXNDC5	Q8NBS9	Thioredoxin domain-containing protein 5	−1.39	0.024
23	Lipid metabolism	ACACB	O00763	Acetyl-CoA carboxylase 2	−1.90	0.044
24		AZGP1	P25311	Zinc-alpha-2-glycoprotein	1.59	0.041
25		PCSK9	Q8NBP7	Proprotein convertase subtilisin/kexin type 9	−1.90	0.003
26		PTGDS	P41222	Prostaglandin-H2 D-isomerase	3.67	0.000
27	Lysosome-based	CTSA	P10619	Lysosomal protective protein	2.75	0.009
28		CTSL	P07711	Cathepsin L1	1.24	0.029
29		DNASE2	O00115	Deoxyribonuclease-2-alpha	2.97	0.033
30		SORT1	Q99523	Sortilin	−5.13	0.005
31	Tissue homeostasis	ABI3BP	Q7Z7G0	Target of Nesh-SH3	5.71	0.008
32		GRN	P28799	Granulins	1.73	0.008
33		MYDGF	Q969H8	Myeloid-derived growth factor	−1.93	0.046
34		NRP1	O14786	Neuropilin-1	1.74	0.029
35		RBP4	P02753	Retinol-binding protein 4	−1.91	0.011
36	Remaining	APP	P05067	Amyloid-beta A4 protein	−1.44	0.033
37		CES1	P23141	Liver carboxylesterase 1	4.05	0.008
38		CHI3L2	Q15782	Chitinase-3-like protein 2	3.43	0.005
39		DMKN	Q6E0U4	Dermokine	−5.40	0.000

NF: normal feeding, GR: glucose restriction, RF: refeeding. FC_(GR+RF)/NF: fold change between GR plus RF (T22RF) and NF (T18). When FC >1, the value was described as FC; otherwise, the value was described as −1/FC.

**Table 4:** Complement factors and cathepsins.

Order	UniProt	Gene Symbol	Description	Category	FC	*p* Value
1	P00736	C1R	Complement C1r subcomponent	Complement factor	−1.00	0.716
2	Q07021	C1Q	Complement C1q subcomponent	Complement factor	−2.96	0.011
3	P09871	C1S	Complement C1s subcomponent	Complement factor	−1.53	0.131
4	P01024	C3	Complement C3	Complement factor	−1.03	0.840
5	P0C0L5	C4B	Complement C4-B	Complement factor	2.85	0.002
6	P00751	CFB	Complement factor B	Complement factor	4.06	0.018
7	P00746	CFD	Complement factor D	Complement factor	−1.93	0.002
8	P08603	CFH	Complement factor H	Complement factor	−1.15	0.277
9	P13987	CD59	CD59 glycoprotein	Complement factor	1.10	0.634
10	O00602	FCN1	Ficolin-1	Complement factor	−4.42	0.578
11	P05155	SERPING1	Plasma protease C1 inhibitor	Complement factor	−1.83	0.190
12	P48740	MASP1	Mannan-binding lectin serine protease 1	Complement factor	2.33	0.196
1	P10619	CTSA	Cathepsin A	Lysosome-based	2.75	0.009
2	P07858	CTSB	Cathepsin B	Lysosome-based	1.19	0.097
3	P07339	CTSD	Cathepsin D	Lysosome-based	1.16	0.309
4	Q9UBX1	CTSF	Cathepsin F	Lysosome-based	1.33	0.239
5	P43235	CTSK	Cathepsin K	Lysosome-based	1.45	NA
6	P07711	CTSL	Cathepsin L1	Lysosome-based	1.24	0.029
7	Q9UBR2	CTSZ	Cathepsin Z	Lysosome-based	1.08	0.466

Fold change (FC) was calculated with the abundance of T22RF/T18. When FC >1, the FC value was described as FC, otherwise the value was described as −1/FC. NA: abundance data available for only one sample. RF: refeeding, NF: normal feeding.

## Supplementary Materials

Supplementary materials can be found at https://www.mdpi.com/1422-0067/20/16/4055/s1, (The file contains Tables S1 and S2).

## Abbreviations

CR	calorie restriction
SGBS	Simpson Golabi Behmel Syndrome
GR	glucose restriction
RF	refeeding
NF	normal feeding
DMEM/F12	Dulbecco’s Modified Eagle Medium: Nutrient Mixture F-12
FC	fold change
C1q	complement factor 1Q
AOC3	membrane primary amine oxidase
CSRP1	cysteine and glycine-rich protein 1
LGALS1	galectin-1
DDT	D-dopachrome decarboxylase
PRSS3	trypsin-3
MT1G	metallothionein-1G
PSMF1	proteasome inhibitor subunit 1
SPRR2G	small proline-rich protein 2G
ENPP2	ectonucleotide pyrophosphatase/phosphodiesterase family member 2
ECM	extracellular matrix
LC-MS/MS	liquid chromatography tandem mass spectrometry
CFB	complement factor B
ADAMTSL1	ADAMTS-like protein 1
ABI3BP	target of Nesh-SH3
CES1	liver carboxylesterase 1
SORT1	sortilin
DMKN	dermokine
PTGDS	prostaglandin-H2 D-isomerase
C4-B	complement factor 4B
CFD	complement factor D
ASP	acylation-stimulating protein
CTSA	cathepsin A
CTSB	cathepsin B
CTSL	cathepsin L
CTSS	cathepsin S
APP	amyloid-beta A4 protein
CHI3L2	chitinase-3-like protein 2
MASP	mannan-binding lectin serine protease
